# Wound healing treatment by high frequency ultrasound, microcurrent, and combined therapy modifies the immune response in rats

**DOI:** 10.1590/bjpt-rbf.2014.0141

**Published:** 2016-01-19

**Authors:** Raciele I. G. Korelo, Marcelo Kryczyk, Carolina Garcia, Katya Naliwaiko, Luiz C. Fernandes

**Affiliations:** 1 Programa de Pós-graduação em Educação Física, Coordenação do Curso de Fisioterapia, Universidade Federal do Paraná (UFPR), Curitiba, PR, Brazil; 2 Faculdade Dom Bosco, Curitiba, PR, Brazil; 3 Departamento de Biologia, UFPR, Curitiba, PR, Brazil; 4 Programa de Pós-graduação em Educação Física, Departamento de Fisiologia, UFPR, Curitiba, PR, Brazil

**Keywords:** wound healing, ultrasonic therapy, electric stimulation therapy, physical therapy specialty

## Abstract

**BACKGROUND:**

Therapeutic high-frequency ultrasound, microcurrent, and a combination of the two have been used as potential interventions in the soft tissue healing process, but little is known about their effect on the immune system.

**OBJECTIVE:**

To evaluate the effects of therapeutic high frequency ultrasound, microcurrent, and the combined therapy of the two on the size of the wound area, peritoneal macrophage function, CD4^+^ and CD8^+^, T lymphocyte populations, and plasma concentration of interleukins (ILs).

**METHOD:**

Sixty-five Wistar rats were randomized into five groups, as follows: uninjured control (C, group 1), lesion and no treatment (L, group 2), lesion treated with ultrasound (LU, group 3), lesion treated with microcurrent (LM, group 4), and lesion treated with combined therapy (LUM, group 5). For groups 3, 4 and 5, treatment was initiated 24 hours after surgery under anesthesia and each group was allocated into three different subgroups (n=5) to allow for the use of the different therapy resources at on days 3, 7 and 14 Photoplanimetry was performed daily. After euthanasia, blood was collected for immune analysis.

**RESULTS:**

Ultrasound increased the phagocytic capacity and the production of nitric oxide by macrophages and induced the reduction of CD4^+^ cells, the CD4^+^/CD8^+^ ratio, and the plasma concentration of IL-1β. Microcurrent and combined therapy decreased the production of superoxide anion, nitric oxide, CD4^+^-positive cells, the CD4^+^/CD8^+^ ratio, and IL-1β concentration.

**CONCLUSIONS:**

Therapeutic high-frequency ultrasound, microcurrent, and combined therapy changed the activity of the innate and adaptive immune system during healing process but did not accelerate the closure of the wound.

## BULLET POINTS

- Ultrasound therapy and microcurrent changed the activity of the innate and adaptive immune system.
- Combined therapy further accelerated the response of the adaptive immune system.
- Ultrasound, microcurrent, and combined therapy did not accelerate the closure of acute wounds.

## Introduction

Wound healing is a process in which the body tissue repairs itself through the coordinated action of extra- and intracellular events^1^. The wound-healing process leads to a formation of new tissue that is structurally and functionally identical to its previous state^2^. Biomarkers of inflammation can be monitored, and their persistence in the blood has been associated with disturbances in the healing process. This source of the inflammation has been attributed to macrophages^3^.

Macrophages are cells from the innate immune system that phagocytose and kill pathogenic organisms^2^; they also produce proinflammatory cytokines (e.g.tumor necrosis factor [TNF]-α, interleukin [IL]-1β, and IL-6) and chemically reactive oxygen and nitrogen molecules. Forty eight hours after tissue injury, macrophages also release IL-10, an anti-inflammatory cytokine which initiates the remodeling of tissues in conjunction with growth factors^4^. Following the migration of macrophages to injured tissue, subsets of lymphocytes do the same. For example, CD4^+^ T lymphocytes are anti-inflammatory while CD8^+^ T lymphocytes are proinflammatory, and the CD4^+^/CD8^+^ ratio is reduced as the healing process progresses^5^.

The management of wound preparation must follow the principle of tissue infection moisture edge (TIME), meaning that the microenvironment must be maintained to support conditions favorable to the healing process^6^. For the treatment of wounds, non-focused high-frequency ultrasound (HFU)^7^^-^ 11 and microcurrent electrical stimulation (MET)^12^^-^ 18 have been used as adjuvant therapies for wound healing. Several studies have reported the positive effects of these approaches based on different mechanisms^7^^,^ 8 ^,^ 10 ^,^ 12 ^,^ 15 ^,^ 16 ^,^ 18 in the wound-healing process. However, the effects of HFU and MET on the immune system are not fully known, particularly in terms of macrophages and subsets of lymphocyte cells.

The combination of these two therapeutic modalities could amplify the effect of one modality alone. Combined therapy in the context of this paper consists of the therapeutic application of an electrical current through the ultrasonic transducer, providing sound pulses and electrical current flow simultaneously. This association saves time in the application and it is believed that the ultrasound increases the permeability of cell membranes, favoring the action of electrical currents on the nervous tissue^19^. However, research is scarce on the benefits of this synergy, and to the authors knowledge, the combined therapy of the two modalities has not been studied in relation to its effect on wound healing. Therefore, it was hypothesized that the use of physical therapy modalities (i.e. HFU and MET) applied alone would promote beneficial changes in the activities of the innate and adaptive immune system, and that the combined therapy would result in further acceleration of the wound-healing process over that which was believed to occur when each modality was applied as a treatment in isolation.

In this study, the effects of HFU, MET, and combined therapy on wound area, peritoneal macrophage function, lymphocyte immune functions, and the plasma concentration of ILs were investigated.

## Method

### Animal model

A total of 65 male Wistar rats were used (7 weeks old, weighing about 312.7 g, standard error of the mean [SEM]=11.6 g). The rats were kept in an environment that had a constant temperature (23±1 °C) under a light/dark cycle (12/12 h), with free access to food and water. This study followed the ethical rules established by Brazilian Law 11.794/08 and the recommendations of the Brazilian College of Animal Experimentation, Moreover, it was approved by the Animal Research Ethics Committee of the Universidade Federal do Paraná (CEUA-UFPR), Curitiba, Paraná, Brazil (protocol number: 561/2012).

### Experimental design

The animals (n=65) were randomly allocated into five groups as follows: control (C, group 1, n=5), lesion and no treatment (L, group 2, n=15), lesion treated with ultrasound therapy (LU, group 3, n=15), lesion treated with microcurrent therapy (LM, group 4, n=15), and lesion treated with combined therapy (LUM, group 5, n=15). With the exception of the control group, all groups were subdivided into three subgroups (n=5 each) according to the number of days of treatment. The treatment was conducted once a day everyday until exsanguinated at 3, 7, and 14 days of treatment. The euthanasia of animals was carried out by decapitation following the ethical rules of the Resolution of the CFMV (Federal Board of Veterinary Medicine) number 1000/12. Two animals of group 2 were lost - during the surgical procedure to inflict the original injury. In order to solve this drawback these two animals were replaced at the time of surgery.

### Surgical procedure

**Figure 1:**
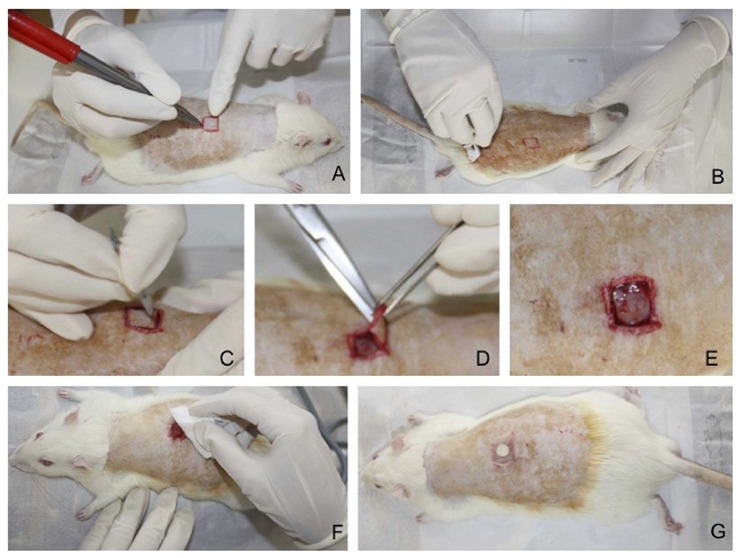
- Surgical procedure for creating the lesion. (A) After hair removal, skin-marking excision wound with plastic mold of 1 cm2 to mark the skin, located on the dorsal medial line of the animal, using 1 cm below the transverse line connecting the lower angle of the scapula as the cranial limit. (B) Asepsis with topical povidone-iodine. (C) Incision with a scalpel blade around the marked tissue. (D) Dissection of the excised skin in the suprafascial plane with tweezers and Mayo scissors, respecting the muscular fascia (2 mm deep). (E) Resection of the skin segment demarcated. (F) Cleaning of excision wound with sterile gauze soaked in saline solution. (G) Primary coverage placement with self-adhesive dressing.

The rats were anesthetized with xylazine (0.05 mL, 10 mg/mL, IM/intramuscular) and ketamine (0.15 mL, 50 mg/mL, IM) diluted in 9% potassium chloride (0.25 mL). After the hair was removed from the back of each rat, the surgical procedure was carried out in which an area of skin was excised by one of the investigators (Figure 1), as described in details elsewhere^20^. After creating the wound, the lesion was cleaned with sterile gauze soaked in saline and received a primary cover (circular self-adhesive dressing of 1.5 cm) maintained for 4 hours after the excision. The animals were kept at room temperature under a warm heating pad to prevent hypothermia until full recovery. The animals were then kept in the room noted above and were allowed to have social interaction with the other four animals of the same subgroup.

### Treatment protocol

**Figure 2:**
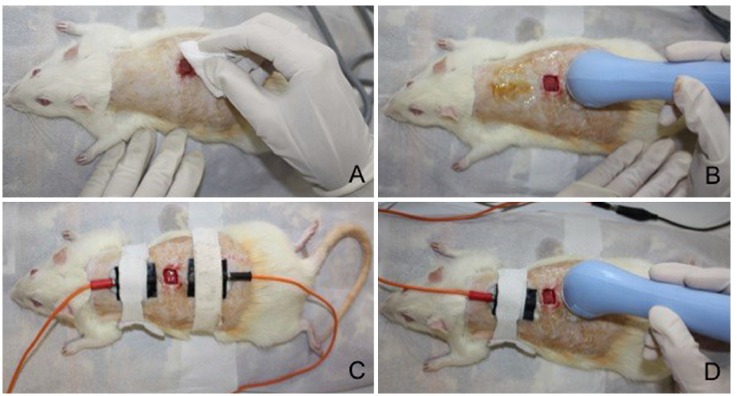
- Intervention procedures in the different groups. (A) Cleaning the wound with sterile gauze and saline solution made for the L group (lesion with no treatment group) and prior to the application of the other three forms of intervention. (B) Application of therapeutic ultrasound in animals from the LU group (lesion treated with ultrasound group). (C) Application of microcurrent in animals from the LM group (lesion treated with microcurrent group). (D) Application of combined therapy in animals from the LUM group (lesion treated with ultrasound and microcurrent group).

The treatment was initiated 24 hours after surgery (Figure 2) and carried out once a day everyday, following the schedule (3, 7, or 14 days), before euthanasia of animals. With the exception of the control group, all groups had their wounds cleaned daily, under anesthesia, prior to the application of the therapeutic resources. This was done with sterile gauze soaked in saline, and primary coverage was not returned after cleaning. The equipment used underwent assessment and received calibration certification. For the dosage, each parameter was chosen based on previous scientific evidence^8^^,^ 13 ^,^ 17. For the LU group (group 3), the HFU (Sonopulse III^(r)^ IBRAMED portable equipment) was set using a transducer at 3 MHz with the following parameters: effective radiating contact area of 3.5 cm^2^; pulsed mode (100 Hz); 50% duty cycle; spatial and temporal average intensity of 1.0 W/cm^2^ and 0.5 W/cm^2^, respectively; and therapeutic dose of 33.6 J/cm^2^. The therapy was carried out for 8 minutes via direct contact with water soluble gel around the wound area (25 cm^2^). For the LM group (group 4), a Neurodyn Esthetic^(r)^ IBRAMED was used to deliver MET. Conductive silicone electrodes (30 mm × 50 mm) with water soluble gel were wrapped around the wound and the following parameters were used: intensity 300 μA, pulse frequency 100 Hz, monophasic rectangular pulse with polarity inversion at every 3 seconds, performed for 8 minutes. For the LUM group (group 5), the two modalities were interconnected using a cable specifically so that the ultrasonic transducer could apply both the sound and electrical pulse. Both modalities were parameterized and the procedures were the same as those described for the LU & LM groups (groups 3 & 4); the application was carried out with a static silicon-carbon electrode positioned at the cranial edge of the lesion. The ultrasonic transducer applied by direct contact by manual sliding the transducer around the free borders of the wound.

### Percentage reduction of wound area-photoplanimetry

Wound contraction was evaluated daily until 14 days after surgery and therapies by photographic documentation. For standardization of images, a fixation system was used for the camera with the distance set at 15 cm; the light was controlled using two cold lamps with a power of 45 W. Evaluation wound contraction was analyzed by measuring the area of wound (transition of regular scar tissue skin on the photo), calculated using ImageJ software 1.47t^15^, calibrated by the caliper used adjacent to the edge of wound^13^, and expressed in percentage. The percentage reduction in the area of the wound was calculated using the following formula^21^:


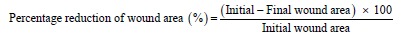


### Interleukin plasma concentration

The measurements of IL-1β, 6, and 10 and TNF-α in the plasma were performed following the instructions of the ELISA kit manufacturer (Boster Immunoleader^(r))^.

### Macrophage immune parameters

Resident macrophages were obtained by intraperitoneal lavage from the groups treated for 14 days to verify the functional parameters. Phagocytic capacity, neutral red retention, superoxide anion, hydrogen peroxide (H_2_O_2_), and nitric oxide (NO) production were performed in quadruplicate as described elsewhere^22^.

### T lymphocyte subpopulations CD4+/CD8+

Flow cytometry was used to measure the presence of CD4^+^ and CD8^+^, markers of helper and cytotoxic T lymphocytes, respectively, on the surface of freshly prepared blood lymphocytes, as has been described elsewhere^23^. About 10^6^ cells/mL in phosphate buffered solution (PBS) supplemented with 0.1% fetal bovine serum (wt/v) and 0.05% sodium azide (wt/v) were incubated for 20 min at 4 °C in the dark with fluorescently labeled monoclonal antibodies to CD4^+^ (clone OX-38) and CD8^+^ (clone OX-8). After staining, cells were washed twice with PBS and immediately examined for fluorescence using a Becton Dickinson FACSCalibur.

### Statistical analysis

Data are presented as mean±SEM and were subjected to normal analysis and homogeneity of variance using the Shapiro-Wilk test and Levene's test, respectively. For analysis of the percentage reduction in wound area, analysis of variance (ANOVA) with repeated mixed measures was used following a design 2 (reduction of percentage wound area: pre vs. post intervention) × 4 (treatment: lesion vs. ultrasound vs. microcurrent vs. combined therapy) × 3 (time: 3 days vs. 7 days vs. 14 days), with a significance level of p<0.05. For the other variables (IL plasma concentration, functional parameters of macrophage and lymphocyte subpopulations), two-way ANOVA between groups was used. Post hoc tests with Bonferroni's correction were employed for multiple comparisons, with a statistical significance of p<0.05. The effect size was determined by calculating omega as follows: ω=0.01 was considered a small effect, ω=0.06 was considered an average effect, and values of ω above 0.14 were considered a large effect. Analyses were performed with IBM-SPSS software version 19 and the graphics prepared with GraphPad PRISM^(r)^ software version 5.0 for Windows.

## Results

### Percentage reduction of wound area

There was a significant reduction in the wound area pre- and post-therapy intervention (Wilks's lambda=0.042, F_1,47_=1076.7, p=0.00, partial eta squared=0.958). In addition, there was a positive interaction between time (3, 7 and 14 days) and pre-and post-intervention in the reduction of wound area (Wilks's lambda=0.096, F_2,47_=221.1, p=0.00, partial eta squared=0.904). There was no significant interaction between the type of treatment and the percentage reduction in wound area pre- and post-intervention (Wilks's lambda=0.881, F_3,47_=2.118, p=0.12, partial eta squared=0.119). Finally, no significant interaction was observed between the types of treatment for percentage reduction in wound area pre- and post-intervention at different times (Wilks's lambda=0.882, F_6,47_=1.047, p=0.41, partial eta squared=0.118).

### Interleukin concentrations

After 3 days (Table 1), only IL-1β was significantly increased (p=0.04 vs. C). The interventions in all three treatment groups (i.e.LU, LM, LUM [groups 3, 4 & 5]) induced a marked reduction in IL-1β (p=0.04, p=0.02, and p=0.04, respectively vs. L). HFU therapy induced a twofold increase in the concentration of IL-6 when compared to the control group (group 1) (p=0.04). At days 7 and 14, the concentration of such cytokines was undetectable.

**Table 1:** - Plasma concentration of interleukin (IL)-1ß, IL-6, IL-10, and tumor necrosis factor (TNF)-a for the experimental period of 3 days after the excision wound model between the different intervention groups (n=5 for each group; C: control; L: lesion without treatment; LU: lesion treated with ultrasound; LM: lesion treated with microcurrent; LUM: lesion treated with combined therapy). The data represent the mean (minimum - maximum), expressed as pg/mL. Each experiment was performed in triplicate.

		C	L		LU		LM		LUM	
IL-1β		nd	45 (27-64)^a^		2.6 (0-37)^b^		nd^b^		13 (6-52)^b^	
TNF-α		nd	nd		nd		nd		nd	
IL-6		nd	3.2 (0.2-6.1)		8.9 (3.5-11)^a^		1.2 (0.1-5.1)		nd^c^	
IL-10		nd	nd		nd		nd		nd	

### Macrophage immune parameters

Table 2 shows the functional parameters of peritoneal macrophages and lymphocytes after surgery at 14 days of therapy. Phagocytosis and neutral red uptake were different between groups (F_4,39_=4.095, p=0.03, ω=0.22 and F_4,39_=7.390, p=0.00, ω=0.38, respectively). Phagocytosis and neutral red uptake in the L group (group 2) were reduced by 47% (p=0.03 vs. C) and 22.5% (p=0.08 vs. C), respectively. The HFU treatment (LU group) [group 3] showed only the recovery of phagocytosis capacity (p=0.04 vs. the lesion without treatment group (group 2)). Neither microcurrent therapy (group 4) nor combined therapy (group 5) reversed the effect caused by excision (p>0.05). Superoxide and hydrogen peroxide production were different between groups (F_4,39_=9.807, p=0.00, ω=0.46 and F_4,39_=25.786, p=0.00, ω=0.78, respectively). Superoxide production did reduce in group 2 (lesion without treatment group) but was not different from the control group (p=0.56) [group 1]. HFU therapy (group 3) caused a further reduction in superoxide production but the reduction was not different from the lesion without treatment group (p=0.72) [group 2]. In contrast, the microcurrent therapy (group 4) and combined therapy (LUM) (group 5) caused a significant reduction (p<0.05 vs. group 2) in the superoxide production (33%) when compared to the Lesion without treatment group (group 2). For hydrogen peroxide, the lesion without treatment group (group 2) showed a reduction of H_2_O_2_ production of 42% (p=0.00 vs. the control group (group 1)). The different modalities (groups 3, 4, & 5) did not modify the H_2_O_2_ production when compared to the Lesion without treatment group (p>0.05) (group 2).

**Table 2:** - Functional parameters from peritoneal macrophages and blood CD4+ and CD8+ T lymphocytes 14 days after excision in the groups (n=5 for each group; C: control; L: lesion with no treatment; LU: lesion treated with ultrasound; LM: lesion treated with microcurrent; LUM: lesion treated with combined therapy). Macrophage data represent mean±SEM, expressed as absorbance/106 cells. Experiment was performed in octuplicate. Data from lymphocytes are expressed as percentage and the experiments were performed in duplicate.

		C			L		LU		LM		LUM	
Macrophages Phagocytic capacity		1.91±0.21			1.0±0.20^^1^^		1.9±0.28^b^		1.74±0.35		1.58±0.24	
Red retention		0.40±0.02			0.31±0.01^a^		0.32±0.01		0.26±0.01^a^		0.29±0.01^a^	
Superoxide anion		2.56±0.19			2.12±0.15		1.71±0.16^a^		1.41±0.75^a,b^		1.44±0.15^a,b^	
Hydrogen peroxide		1.60±0.07			0.93±0.06^a^		0.85±0.03^a^		1.09±0.08^a^		0.82±0.03^a,c^	
Lymphocytes T CD4^+^		21.2±0.4			22.6±0.2^a^		19.0±0.2^a,b^		19.4±0.3^a,b^		12.0±1.9^a,b,c,d^	
T CD8^+^		19.0±0.2			14.9±0.3^a^		19.0±1.9		20.3±0.8^b^		17.6±5.8	
TCD4^+^/CD8^+^		1.1±0.04			1.5±0.01^a^		1.0±0.11^b^		0.9±0.05^b^		0.8±0.17^b^	

**Figure 3:**
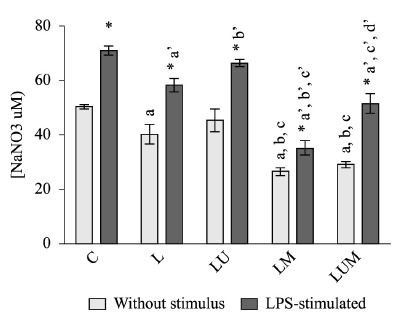
- Nitric oxide production by peritoneal macrophages 14 days after excision model without stimulus and LPS-stimulated in the groups (C: control; L: lesion with no treatment; LU: lesion treated with ultrasound; LM: lesion treated with microcurrent; LUM: lesion treated with combined therapy). The data represent the mean±SEM, expressed as µmol/L. Each experiment was performed in quadruplicate. *p

The production of NO is shown in Figure 3. This was different between groups (F_4,39_=15.451, p=0.00, ω=0.74). The basal concentration of NO production by peritoneal macrophages was reduced in the lesion without treatment group (group 2) (p=0.04 vs. the control group (group 1)). HFU therapy (group 3) did reverse the effect of the lesion (p=0.39 vs. the control group [group 1]). In contrast, for the microcurrent therapy alone group (LM group or group 4) and in combination with the HFU (the LUM group or group 5), the production was about 50% of the basal state. Under lipopolysaccharide (LPS) stimulation, it was also statistical different between the groups (F_4,19_=30.875, p=0.00, ω=0.85). Under LPS stimulation, peritoneal macrophages increased in about 30% from control (p=0.00 *vs* non-stimulated). The same was seen in the L (group 2) and LU (group 3) groups (p<0.01 *vs.* non-stimulated). In the LM group (group 4), LPS stimulation increased the nitric oxide production by ~20% (p=0.02 *vs.* non-stimulated). In the presence of LPS, the combined therapy (group 5) increased the NO production by ~40% (p<0.01 *vs.* non-stimulated).

### T lymphocyte subpopulations CD4+/CD8+

The excised lesion (group 2) increased (Table 2) the CD4^+^ T lymphocyte subset (6.19%) as compared to control group (group 1) (p=0.03). The therapy with HFU (group 3) or microcurrent (group 4) significantly decreased (~15%) the population of CD4^+^ T lymphocytes when compared to the Control (group 1) and L (group 2) groups (p=0.02 and p=0.04, respectively). The combined therapy (group 5) showed an even further reduction 45%; (p=0.04 vs. control (group 1), p=0.03 vs. lesion without treatment (group 2), p=0.02 vs. LU (group 3) and p=0.02 vs. LM (group 4)). CD8^+^ T lymphocytes in the lesion group (group 2) was reduced by 22% (p=0.02 vs. control group (group 1)). The different therapies (groups 3 & 4) and their combination (group 5) recovered the CD8+ T lymphocyte population to control levels (p>0.05 vs. control group (group 1)). The CD4^+^/CD8^+^ ratio increased in the lesion group (group 2) (p=0.03 vs. control group (group 1)). The different therapies (groups 3 & 4) and their combination (group 5) reversed the effects caused by the excision lesion to control level (p=0.04 vs. LU (group 3), p=0.03 vs. LM (group 4), p=0.02 vs. LUM (group 5)).

## Discussion

In this study, the effect of HFU, microcurrent, and combined therapies on the immune system and healing process in a wound-induced excision model was investigated. Contrary to the findings of this study, other studies have shown acceleration of the wound-healing process with HFU^8^^,^ 10 ^,^ 20 and MET^12^^,^ 16; however, this previous research did not explore the immune system as was done in the present study. Inflammation is a key factor in the healing process, followed by cell proliferation and maturation^2^^-^ 4. Our results showed that the two different physical therapy modalities were able to significantly modulate macrophages immune parameters, decrease the expression of pro-inflammatory cytokines, and decrease the expression of CD4^+^-positive cells in association with a reduced CD4^+^/CD8^+^ ratio. In addition, the combination of both modalities (combined therapy) further decreased the expression of CD4^+^-positive cells and the CD4^+^/CD8^+^ ratio. These changes, which were brought about by the combined therapy, suggest that the rats immune system was attempting to solve the healing process. However, although the different approaches resulted in a significant reduction in the wound area, their use for this purpose was not supported, as there was no statistically significant difference compared to untreated excised lesions.

Because these therapies were able to change the immune parameters, helping to modulate the healing process, and given the fine line of percentage change between the different interventions, a change in wound area would also be expected. However, the present study did not detect such a result. This result might have been affected by the dosage parameters of the modalities used, although based on previous evidence^8^^,^ 13 ^,^ 17, the dosage used in this study was not enough to promote the acceleration of wound healing. It must be pointed out that in the present study, only a specific dosages were evaluated; thus, the results cannot be extrapolated to conditions in which other parameters and/or different forms of electrical stimulation may be used. Further research is needed to determine the ideal dose-response treatment for the different stages of wound healing.

It has been shown^24^ that different doses of HFU and microcurrents increase the tensile strength of tissue being repaired, which is important for tissue quality. As a matter of fact, the cytokine network is important for tissue quality^2^^,^ 4; the present study showed that different physical therapy modalities are able to alter the plasma concentrations of ILs.

HFU reduced the concentration of IL-1β (known as pro-inflammatory) along with an increase in IL-6 (in the early phase of inflammation, this is a chemoattractant cytokine for keratinocytes) 3 days after the lesion was generated. This was seen with both modalities for IL-1β concentration. The concentrations of IL-6 and TNF-α increased markedly and IL-1β and IL-10 were observed in lower amounts^24^. The methods used in this study somehow contributed to the delicate balance between these cytokines, attenuating the inflammatory response because IL-1β is a potent inducer of the transduction signaling cascade for growth factors involved in the migration of inflammatory cells and the production of prostaglandins for nociception^25^.

An in vitro^26^ study showed that HFU therapy stimulated the release of IL-1β at a low rate due to three possible mechanisms: an increase in cell membrane permeability; changes in the signal transduction that regulated gene expression; and alterations in the cytoskeleton, affecting cell metabolism and gene expression. However, the precise mechanism is still unknown. The reduction of IL-1β by the microcurrent therapy may have lead to the possible involvement of nuclear factor κB (NFκB) and mitogen-activated protein kinase (MAPK)^27^.

Fourteen days after wound-induced excision, HFU increased the phagocytosis capacity and the NO production in the presence of the challenger LPS. The authors are not aware of any study that investigated the innate immune system following physical therapy treatment and the authors hypothesize that this increase was due to the capacity of the ultrasound therapy to induce a stable cavitation related to the transitory increment of cell permeability to calcium influx^7^^,^ 11. Along with increased phagocytic capacity, HFU also increased NO production in the presence of LPS at the same level of control group. This increase may have been caused by higher intracellular calcium concentration^28^^,^ 29, revealing its antioxidant potential and resulted in low levels of the other two redox molecules (i.e. superoxide anion and H_2_O_2_). Indeed, the formation of redox by ultrasound is related to the sonolysis of water generated by the cavitation^11^; however, the acoustic parameters used in the present experiment were insufficient to induce their formation, perhaps because ultrasonic frequencies up to 2 MHz were not able to sonolyze water^30^.

Interestingly, the microcurrent decreased superoxide anion production concurrently with increased production of NO in the presence of LPS, confirming its antimicrobial power, but with lower expression when compared to the lesion without treatment group (group 2). An excess of redox molecules can damage tissues and amplify the pro-inflammatory response, perhaps leading to a chronic stage^31^. Microcurrent therapy increases the adenosine triphosphate (ATP) concentration, where ATP acts as an antioxidant to stabilize mitochondrial function^32^. In fact, low-grade NO production at the end of the healing process restores collagen concentration to physiological levels, but the mechanisms for this are not known^33^. Curiously, combined therapy reduced H_2_O_2_, superoxide anion, and NO production. NO production has been proposed as a common mechanism for both therapeutic approaches^34^, however, this still needs to be proven. The present data does not support this suggestion. The authors think that different energy forms-whether mechanical or electrical-trigger a sequence of events to stimulate or inhibit cell processes which leads to the wound-healing process.

Finally, both modalities alone and in combination reduced the CD4^+^/CD8^+^ ratio, suggesting the resolution of the inflammatory process^5^. The CD8^+^ T cell population was maintained at control levels, resulting in better tissue quality, although this was only applicable to microcurrent therapy because CD4^+^ cells are considered up-regulatory, leading to the formation of fragile scar tissue and CD8^+^ down-regulation, which makes the scar tissue more denserigid^5^. In addition, CD8^+^ cells regulate the production of cytokines through CD4^+^, and consequently, result in a lower level of cytokines^5^. Different organisms with high regenerative capacity (e.g. zebrafish, salamander, and human fetuses) have lower power for stimuli to activate the signaling cascade of the healing process and present with an absence of inflammatory cells at the site of injury, which might be a prerequisite for better repair and tissue quality and/or complete regeneration^3^. This suggests that the modalities used in this study could be useful in modulating the immune system, thereby helping in the formation of a new tissue.

In future studies, the quality of scar tissue should also be measured and local biomarkers should be investigated. In the present study, only circulating markers were measured because the aim of the study was to look at the healing phenomenon from a systemic perspective. The present work sheds light on the therapeutic approaches and the effect of two modalities used alone or together on the immune system. An environment of excessive inflammation may lead to inadequate healing, thereby delaying the healing process and increasing the amount of scar tissue. This means that it might be feasible to manipulate the immune response in the healing process, especially if the patient was willing to sacrifice the wound area reduction rate for a better esthetic result. The physical therapy modalities studied here could contribute to such applications.

## Conclusions

High frequency ultrasound, microcurrent and combining the two modalities were able to modulate the activity of the innate and adaptive immune system, improving the inflammatory environment but not accelerating the wound-healing process. More studies are needed to understand the mechanisms involved in the modulation of wound healing using different physical agents, as well as the relation of these different physical modalities with the quality of the newly formed tissue.
